# myBeST—A Web-Based Survival Prognostic Tool for Women with Breast Cancer in Malaysia: Development Process and Preliminary Validation Study

**DOI:** 10.3390/ijerph20042985

**Published:** 2023-02-08

**Authors:** Mohd Nasrullah Nik Ab Kadir, Suhaily Mohd Hairon, Najib Majdi Yaacob, Siti Norbayah Yusof, Kamarul Imran Musa, Maya Mazuwin Yahya, Seoparjoo Azmel Mohd Isa, Muhammad Hafizuddin Mamat Azlan, Imi Sairi Ab Hadi

**Affiliations:** 1 Department of Community Medicine, School of Medical Sciences, Universiti Sains Malaysia, Kubang Kerian 16150, Kelantan, Malaysia; 2 Biostatistics and Research Methodology Unit, School of Medical Sciences, Universiti Sains Malaysia, Kubang Kerian 16150, Kelantan, Malaysia; 3 Malaysian National Cancer Registry Department, National Cancer Institute, Ministry of Health Malaysia, Putrajaya 62250, Federal Territory of Putrajaya, Malaysia; 4 Department of Surgery, School of Medical Sciences, Universiti Sains Malaysia, Kubang Kerian 16150, Kelantan, Malaysia; 5 Department of Pathology, School of Medical Sciences, Universiti Sains Malaysia, Kubang Kerian 16150, Kelantan, Malaysia; 6 Hospital Sibu, Ministry of Health Malaysia, Sibu 96000, Sarawak, Malaysia; 7 Breast and Endocrine Surgery Unit, Department of Surgery, Hospital Raja Perempuan Zainab II, Ministry of Health Malaysia, Kota Bharu 15586, Kelantan, Malaysia

**Keywords:** breast cancer, prognosis, eHealth, web-based tool, Malaysian women

## Abstract

Women with breast cancer are keen to know their predicted survival. We developed a new prognostic model for women with breast cancer in Malaysia. Using the model, this study aimed to design the user interface and develop the contents of a web-based prognostic tool for the care provider to convey survival estimates. We employed an iterative website development process which includes: (1) an initial development stage informed by reviewing existing tools and deliberation among breast surgeons and epidemiologists, (2) content validation and feedback by medical specialists, and (3) face validation and end-user feedback among medical officers. Several iterative prototypes were produced and improved based on the feedback. The experts (n = 8) highly agreed on the website content and predictors for survival with content validity indices ≥ 0.88. Users (n = 20) scored face validity indices of more than 0.90. They expressed favourable responses. The tool, named Malaysian Breast cancer Survival prognostic Tool (myBeST), is accessible online. The tool estimates an individualised five-year survival prediction probability. Accompanying contents were included to explain the tool’s aim, target user, and development process. The tool could act as an additional tool to provide evidence-based and personalised breast cancer outcomes.

## 1. Introduction

More than 21,000 breast cancer patients were diagnosed within the five years between 2012 and 2016, making breast cancer the most common cancer in Malaysia [1]. Survival probabilities were rated as the most important answers that each newly diagnosed patient seeks to ask healthcare providers [2]. It was a must-have component in developing a mobile application for breast cancer awareness in Malaysia [3].

Personalised breast cancer survival prediction based on prognostic models was commonly presented as regression formula, online tools, decision trees, nomograms, or score charts [4]. With the advent of ubiquitous internet-enabled mobile devices, deploying the prognostic models as an online tool has become widespread [4,5,6]. In addition, the accessibility of the tools enabled independent researchers to evaluate, validate, and provide feedback for improvement. A scoping review revealed 21 unique tools to aid breast cancer treatment decisions [7]. Some of them were related to the survival outcome, such as PREDICT breast cancer [8], CancerMath [9], Adjuvant! Online [10], and Nottingham Prognostic Index [11]. There were other web-based tools for estimating survival, such as Surveillance, Epidemiology, and End Results Cancer Survival Calculator (SEER*CSC) [12], Proview [13], and InterPreT [14].

The PREDICT breast cancer (PREDICT) web-based tool has worldwide access and has increased in popularity [8]. It has been validated in several independent populations, albeit with several limitations [15,16,17,18,19]. The tool was less accurate in predicting breast cancer survival among Malaysian women, especially those younger than 40 years old and receiving neoadjuvant treatment [18]. Adjuvant! Online and CancerMath echoed similar findings with overestimations of survival prediction when validated among women with breast cancer in Malaysia [20,21].

eHealth or electronic health technology, including web-based cancer prognostic tools, should always follow user-centred design principles for more significant usage and successful implementation in clinical practice. The user-centred design principle is a concept in which the end-users and stakeholders participate and influence the eHealth development process from the beginning to the post-implementation stage [22,23].

The development process was usually conducted iteratively, as the product was updated at each stage according to the participants’ feedback. In each stage, various methods such as literature reviews, questionnaires, interviews, or focus groups could be used according to the developer’s feasibility and the product’s needs [22,24]. The iterative development process is one of the Software Development Life Cycle (SDLC) models. It was commonly used for web-based application development. For example, PREDICT breast cancer and SEER*CSC followed user-centred design principles and were developed iteratively [12,25].

To the best of our knowledge, there was no available specific online individualised survival prognostic calculator for women with breast cancer in Malaysia. Due to the limitations of the aforementioned tools derived from the Western population [18,20,21], we have developed a locally adapted prognostic model. The model’s algorithm showed robust performance (c-index: 0.81, integrated Brier score: 0.122, and area under the receiver operating characteristics curve: 0.891) [26].

Therefore, the model’s algorithm was used as the main element of this study to describe the development process and preliminary validation of a web-based prognostic tool, the Malaysian Breast cancer Survival prognostic Tool (myBeST). The resulting tool could potentially equip healthcare providers with an additional reference for risk communication towards patients and caregivers.

## 2. Materials and Methods

The web-based tool was intended to be used by healthcare providers when women with breast cancer ask about their prognosis, specifically a survival probability at five-year. We did not propose the tool to be used directly by patients. Newly diagnosed cancer patients using prognostic tools without professional supervision could lead to inaccurate understanding and emotional distress [27,28].

**Figure 1:**
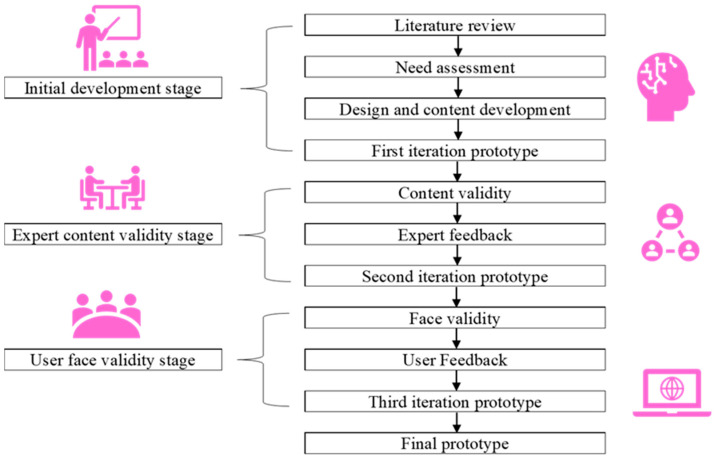
The development process of a web-based prognostic tool (myBeST) for healthcare providers.

We developed our website’s content and user interface using an iterative user-centred design approach [22,24,29] which gathered input from subject matter experts and potential end-users to improve and finalise the prototype’s design and content. In our study, we recruited medical specialists and medical officers at every stage of website development. They were mainly from three tertiary medical centres in Malaysia. The study was conducted between April 2022 and September 2022. The process was divided into three stages: (1) the initial development stage, (2) the expert content validity stage, and (3) the user face validity stage. Figure 1 summarises the development process.

### 2.1. Initial Development Stage

#### 2.1.1. Literature Review

We reviewed existing web-based breast cancer survival prognostic tools and related literature to guide the development process. On top of that, we focused on the frequently used online tools and their development process.

#### 2.1.2. Need Assessment

We conducted a need assessment informed by a literature review and stakeholders’ discussions. The discussion involved breast surgeons, the head of the national cancer registry, and breast cancer researchers (among epidemiologists) as research team members (n = 5).

#### 2.1.3. Design and Content Development

Literature review and existing prognostic tools informed our website’s contents, wording, and design. These elements were further refined by a serial round of discussions among research team members. The sketches of the initial draft of the website were created at the end of this step.

#### 2.1.4. First Iteration Prototype

Input obtained from the previous steps was utilised to develop and deploy the website. The model’s algorithm [26] formed part of the backend code and was deployed as a functioning survival prediction tool (i.e., front-end or user interface) using “Shiny”, an R software package. Subsequently, the tool was embedded in a WordPress template website. The tool’s functionality, navigation of the website, and aesthetics were reviewed and tested by the same research team members prior to the evaluation of the first iteration prototype in the next stage.

### 2.2. Expert Content Validity Stage

We invited eight other independent experts to review the website and prognostic tool. They consisted of a breast surgeon (n = 1), general surgeons (n = 4), and public health medicine specialists (n = 3) with previous research related to breast cancer. The review aimed to obtain overall feedback and collective agreement regarding the predictors used to estimate breast cancer survival and the accompanying website content.

We explained the website’s contents to each expert and their expected task via virtual or face-to-face meetings. The experts were given a self-administered proforma and the website’s uniform resource locator (URL). The proforma consisted of three parts. Part 1 involved the 13 predictors selected for the development of the prognostic tool model. Part 2 focused on the relevancy of the website’s content. Part 1 and Part 2 comprised the content validity component, whereas Part 3 was open-ended feedback regarding the web-based prognostic tool and suggestions to improve it. Two weeks were given to the experts to use the website and return the proforma.

#### 2.2.1. Content Validity

The content validation was analysed using the item-content validity index (I-CVI). Each item was reviewed according to a Likert scale of ‘1’ to ‘4’, with ‘1’ as irrelevant, whereas ‘4’ indicated highly relevant predictors or website content. A four-point Likert scale was used as a measurement scale instead of a five-point or three-point scale to avoid a neutral or ambivalent midpoint. Score 1 was given to ‘3’ and ‘4’. Meanwhile, score 0 was for ‘1’ and ‘2’. The total score was divided by the total number of experts. Thus, the values of I-CVI range between 0 and 1. The scale-level content validity index averaging method (S-CVI/Ave) was calculated by dividing the summation of all the I-CVI scores by the number of items [30,31].

#### 2.2.2. Expert Feedback

We sought expert open-ended feedback using Part 3 of the proforma. The experts were contacted again to clarify their feedback and general comments on the website.

#### 2.2.3. Second Iteration Prototype

Input from the experts at this stage influenced the modifications of the first iterative prototype. Relevant and feasible recommendations were incorporated into the improved second iterative prototype.

### 2.3. User Face Validity Stage

In this stage, we recruited those with working experience in the surgery and oncology department as medical officers (n = 14). They are usually the first person to meet newly diagnosed breast cancer patients in clinical settings headed by the breast (or general) surgeon or oncologist. We also invited medical officers from other medical disciplines (n = 6) to broaden the potential usage of the web-based prognostic tool. The focus at this stage was to determine whether the target end-user understood the website’s content and scored the appropriateness of the user interface.

Similar to the previous stage, a brief overview of the website and a self-administered proforma with a URL were given to each participant. They were asked to complete their assessment within two weeks.

#### 2.3.1. Face Validity

Clarity, understanding, and appropriateness of the website presentation formed the elements of the face validity. A Likert scale of ‘1’ to ‘4’ was used to indicate ‘1’ for strongly disagree and ‘4’ for strongly agree. Subsequently, the scale was dichotomized into ‘0’ or ‘1’ in a similar pattern as in content validation to calculate the face validation index (FVI) score [32].

#### 2.3.2. User Feedback

Additionally, we asked the medical officers about the overall structure and ways to improve the website. Clarifications of their opinions were also sought.

#### 2.3.3. Third Iteration Prototype

In this step, feedback that was relevant and feasible was implemented. The third iteration prototype was deployed for final deliberation among research team members.

### 2.4. Final Prototype

The website was reviewed and tested again to ensure correct content and its intended functionality. Finally, the final product was deployed with an accessible URL.

### 2.5. Ethical Approval

The Medical Research and Ethics Committee, Ministry of Health Malaysia (NMRR-21-37-57989 (IIR)), and the Human Research and Ethics Committee, Universiti Sains Malaysia (USM/JEPeM/21010112) granted us ethical approval. All participants were provided with written, informed consent. We ensured the confidentiality of the participant’s data and the responses were anonymous. Only the authors had access to the data.

## 3. Results

### 3.1. Initial Development Stage

#### 3.1.1. Literature Review

Several reviews cited PREDICT breast cancer as the most commonly used online tool for breast cancer survival estimates [4,5,7]. Thus, PREDICT (https://breast.predict.nhs.uk/ accessed on 3 April 2022) acted as a primary reference or template for developing our web-based prognostic tool.

In addition, recommendations from the developing PREDICT website [25] and SEER*CSC [12] were primarily consulted to develop our web-based prognostic tool. Principal recommendations pertinent to our website development include:

- Involvement of target users and stakeholders: In our case, all the research team members and participants in every stage of the development process were healthcare professionals;
- Simple and various presentations of the survival prediction outputs.

#### 3.1.2. Need Assessment

Based on the literature review, individualised survival was pivotal in managing patients’ expectations regarding their health trajectory and guiding care plans [2,3]. On top of that, discussions with stakeholders advocated for an online prognostic tool employing local datasets. Stakeholders depended on other countries’ tools or overall tumour node metastasis (TNM) stage survival estimates with heterogenous findings [33,34,35,36]. They viewed the tool as a promising element of risk communication during patient encounters.

#### 3.1.3. Design and Content Development

Research team members agreed to include the website content summarised in Table 1. In addition, it was recommended that the website is accessible across multiple platforms by using browsers on any mobile device and desktop computer. Based on the recommendations, the website needs to be simple and concise to achieve an exemplary user interface.

**Figure 2:**
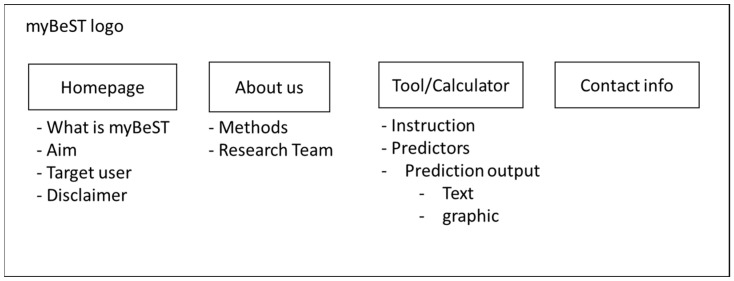
Sketches of the website’s initial draft.

The website’s main feature is individualised survival prediction. The prediction was based on the previously-developed prognostic model [26]. The prediction model aimed to provide personalised five-year survival probability predictions based on women with breast cancer in Malaysia. As suggested by previous studies, we opted to present the survival probability in text and graphic visualisation format for better comprehension [28,37]. These reviews implied that risk information, such as percentages, should be presented clearly and precisely. A simple graph with a limited amount of accompanying information was recommended. Sketches of the initial draft of the website are shown in Figure 2.

#### 3.1.4. First Iteration Prototype

The first iteration prototype website consisted of the written statement of the items described in Table 1. For the prognostic tool on the “Tool” page, the model’s algorithm calculated the personalised survival at five years depending on the 13 variables’ input. These variables include demographic parameters, cancer characteristics, and treatment receipt. No individual data were collected or stored on the server’s website as we only deployed the model’s algorithm into the server.

### 3.2. Expert Content Validity Stage

#### 3.2.1. Content Validity

The expert panel for content validity responded positively towards the web-based prognostic tool. They have a median (IQR) of 14.0 (12.0–18.0) years of working experience. I-CVI values of all predictors (Table 2) ranged between 0.88 and 1.00, whereas the S-CVI was 0.97, indicating the relevancy of the 13 predictors included in the prognostic tool.

Likewise, eight experts agreed on the relevancy of all elements incorporated in the website content with an S-CVI/Ave value of 1.00, as all the I-CVI values of each content heading were 1.00. The detailed result is in Table 3.

#### 3.2.2. Expert Feedback

An expert suggested the inclusion of additional predictors such as Ki-67, menopausal status, and targeted therapy such as trastuzumab for future versions. Another expert commented that treatment such as local chest radiotherapy might not be recommended for women with advanced breast cancer. The inclusion of the tool’s limitations and publications describing the methodology and validity of the model’s algorithm were suggested by another specialist. Regarding design, there were suggestions to display the prediction tool on the homepage and deploy it in a mobile application format.

#### 3.2.3. Second Iteration Prototype

Following expert review, the website’s content and tool’s predictors were kept with adjustments to the wording to provide clarity of the content. New predictors could not be added as the original prognostic model did not include the suggested predictors. We added a link to the recently published article describing the model’s development [26]. We kept the prognostic tool on a separate page with a link on the front page to accustom the user to the aim, target use, overview, and limitations of the web-based prognostic tool. We kept the suggestion for the inclusion of other predictors and deployment in the mobile application for future work.

### 3.3. User Face Validity Stage

#### 3.3.1. Face Validity

A total of 20 target users among medical officers participated in the face validation study. They had a median (IQR) working experience of 9.5 (9.0–11.0) years. The result showed favourable face validity with all FVI elements rated more than 0.90, as in Table 4.

#### 3.3.2. User Feedback

One participant gave a lower rating for the input predictors. He/she and other users suggested clear definitions of predictors such as tumour grade classification, staging, and early definitive surgery. Open-ended feedback demonstrates that users have a positive attitude towards the website. They commented that the tool is easy to use, understandable, and straightforward. Similar to the comments made by experts, additional predictors such as targeted therapy and hormonal therapy were suggested for a future update.

#### 3.3.3. Third Iteration Prototype

After several discussions among research team members, minor adjustments were made to provide a clear definition of each predictor. Unfortunately, we could not include new predictors due to the limitations of the original prognostic model. The resulting final product website containing the prognostic tool is described in the following sections.

### 3.4. Final Prototype

#### 3.4.1. Website—myBeST

**Figure 3:**
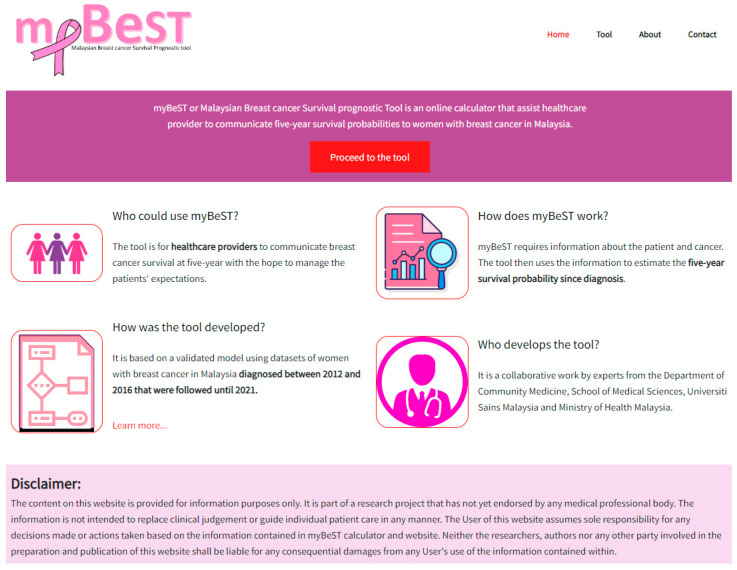
Homepage of the website.

**Figure 4:**
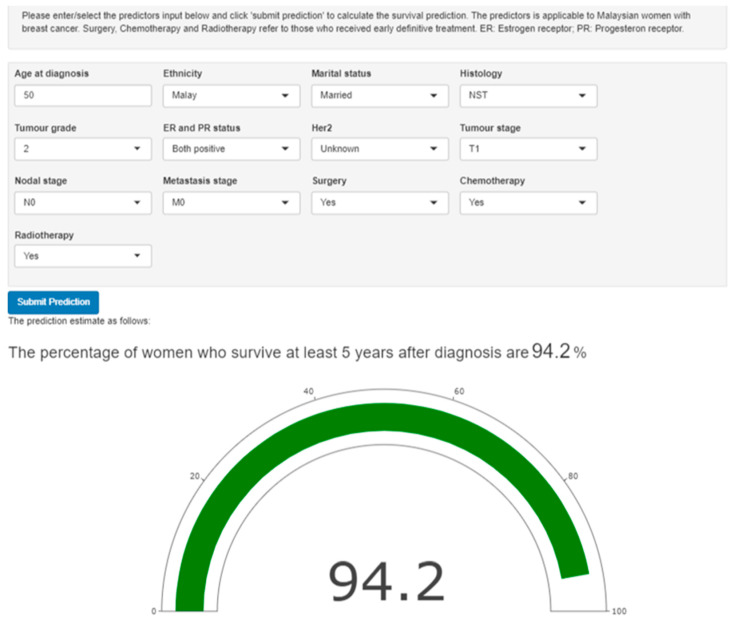
A screenshot of Shiny interface to predict survival probability among women with breast cancer in Malaysia.

**Figure A1:**
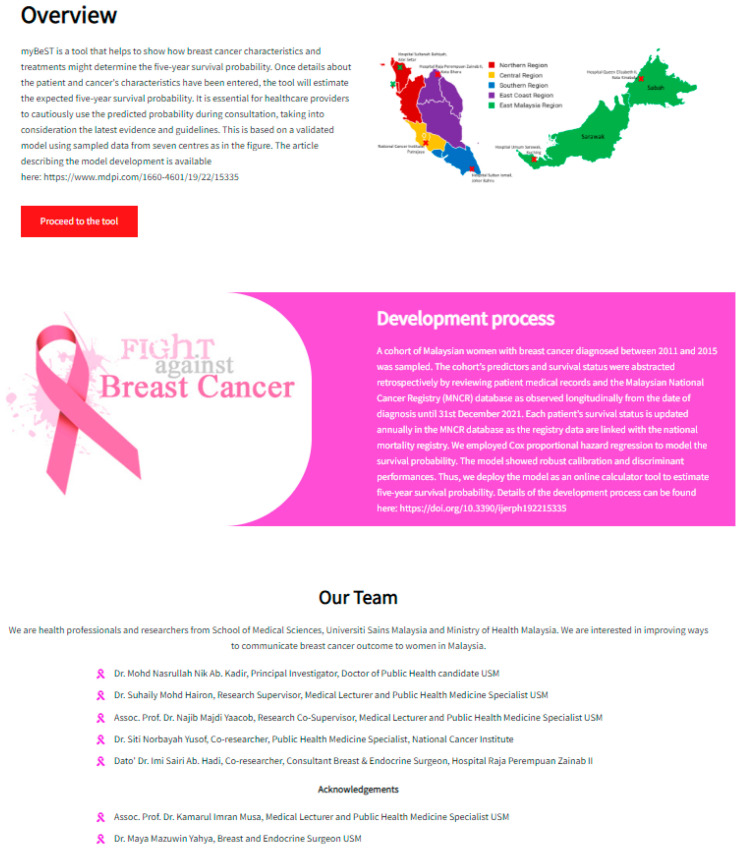
About page of the website.

We named the web-based prognostic tool Malaysian Breast cancer Survival prognostic Tool, abbreviated as myBeST. The website can be accessed via the URL: http://mybestpredict.com/ (accessed on 21 September 2022). The website contains four pages, namely, the homepage (Figure 3), tool page (Figure 4), about page (Figure A1), and contact page. Headings and descriptions of each content on the website are shown in Table 1.

#### 3.4.2. The Web-Based Prediction Tool

The tool was designed using the R Shiny web interface (Figure 4) and deployed in the cloud-based server, https://www.shinyapps.io/ (accessed on 2 April 2022). The tool consists of three components: (1) instruction, (2) input predictors, and (3) prediction outputs. The instruction describes how to use the tool and the definition of the predictors’ input. The second component requires users to enter (age at diagnosis variable) or select input with drop-down options necessary to calculate the prediction. The prediction output component of the survival probability is displayed in two ways: (1) by the text of the five-year survival probability percentage and (2) by a meter gauge graph to visualise survival probability. The entered patient’s parameter is not stored in our database. For example (Figure 4), a 50-year-old Malay married woman with grade II, both ER and PR positive, invasive carcinoma (NST) diagnosed at T1, N0, M0 stage, who received surgery, chemotherapy, and radiotherapy has a 94.2% percentage to survive at least five years after diagnosis.

## 4. Discussion

We developed the content and user interface of a web-based prognostic tool for healthcare providers to convey disease trajectory among Malaysian women newly diagnosed with breast cancer. It enables care practitioners to provide individualised five-year survival probability once the predictors are entered into the online tool. To the best of our knowledge, this study resulted in the first web-based prognostic tool to inform women with breast cancer in Malaysia.

As any instrument or tool requires some form of validation for the intended user, the focus of our current paper was the content and face validity of the model presented as a web-based prognostic tool. The validity of the model algorithm has been described in our previous paper [26]. Preliminary validity assessments showed encouraging responses from the medical specialists and medical officers. Expert content validation is crucial to ensure that only relevant predictors and website content are included. I-CVI of more than 0.83 would indicate agreement content validation if the expert panel were comprised of eight or more individuals [30,31]. The minimum value recommended for S-CVI/Ave is 0.83 [30,31]. Although the result was satisfactory, the experts had several suggestions to improve the website. Before commencing the face validity study, we had implemented several feasible suggestions by the experts.

For face validity, a higher FVI of more than 0.80 indicates an acceptable result [38,39,40]. The feedback from the medical officers was, more or less, similar to that of the experts in the previous stage. Thus, we implemented several additional feasible improvements. Their feedback as end-users was valuable for improving the website as an additional tool for risk communication.

Despite the positive feedback, implementing prognostic tools in clinical settings remains elusive [12]. It could be due to a multitude of reasons, such as individual clinician preference, short consultation time, and heterogeneity of attitude and literacy among women with breast cancer [25,27,28,37]. In addition, incorporating or embedding prognostic tools in electronic medical records could reduce the hassle of re-entering data and potentially improve usage during the consultation [41].

A strength of the tool is that it is in a web-based format instead of a mobile application to increase the accessibility of using it on a desktop computer and mobile devices that always have an internet browser. In addition, presenting survival information in a web-based format allows for accessibility and dissemination of the information.

Secondly, we sought to include established routinely collected predictors for wide adaption in limited-resource settings. The inclusion of ethnicity as a predictor was crucial to reflect substantially different mortality risks in our setting. It differentiated our tool from the existing Western-centric prognostic tools. The website was also designed with a simple user-centred approach. Practical prognostic tools must be readily available, low complexity, and easy to use. It could be used as a component during health education and clinical consultation. We also included an article describing the underlying model data and modelling procedure that could improve clinician trust in the prognostic tool [27].

There are some limitations of our study. Firstly, the website requires an internet connection. However, a breast cancer diagnosis is usually made at a main medical centre in an urban area with good internet coverage. Secondly, users should be cautious when applying the tool as the model needs further evaluation with an independent dataset. Finally, similar to other clinical prediction models/tools, patient-facing use should only be applied when there are no clear-cut benefits against harm or while influencing patients’ hesitancy towards evidence-based intervention.

Other limitations include factors such as targeted therapy and hormonal therapy that were not accounted for in the model’s algorithm. Several experts and medical officers considered these predictors necessary in survival prediction estimation. However, the model only considered predictors at baseline and was constrained to the limited follow-up time based on the medical records’ documentation.

Future works should consider the implementation of the remaining suggestions of the experts and users. Naturally, upcoming research would involve feasibility, usability, and acceptability study at the patient-facing consultation level. Conducting the study with a larger and more diverse sample of healthcare providers would be essential to improving the website, particularly the tool’s clinical and social implications. Website development is an iterative process that needs to be updated as new requirements become necessary.

## 5. Conclusions

In summary, the developed web-based prognostic tool showed positive preliminary evaluation by content and face validity. Healthcare providers might use the web-based prognostic tool as an adjunct to communicate five-year survival among women with breast cancer in Malaysia. Future research and updates must be conducted to ensure effective implementation and usage.

## Figures and Tables

**Table 1:** The website pages and contents’ description.

Page	Heading	Content
Home	myBeST header	Definition and aim of the tool
	Who could use myBeST?	Target user: healthcare provider
	How does myBeST work	Requirement and output of the tool
	How was the tool developed?	Description of the model development
	Who developed the tool?	Researchers and their background
	Disclaimer	Disclaimer of using the tool
Tool	Tool	Embedded R Shiny interface of the prediction tool
About	Overview	The aim, usage, and limitation of the tool
	Development process	Cohort description and analysis used
	Our Team	Researchers involved in the studies
Contact	Contact	Feedback form and location of the researchers

**Table 2:** The item-content validation index (I-CVI) for each predictor and scale-level content validity index, averaging method (S-CVI/Ave) of the prognostic tool by experts panel (n = 8).

Item (Predictors)	Total Score	I-CVI
Age at diagnosis	7	0.88
Ethnicity	7	0.88
Marital status	7	0.88
Histology	8	1.00
Tumour grade	8	1.00
ER and PR status	8	1.00
HER2	8	1.00
Tumour stage	8	1.00
Nodal stage	8	1.00
Metastasis stage	8	1.00
Surgery	8	1.00
Chemotherapy	8	1.00
Radiotherapy	8	1.00
	S-CVI/Ave	0.97

ER: Oestrogen receptor; PR: progesterone receptor; HER2: human epidermal growth factor receptor 2.

**Table 3:** The item-content validation index (I-CVI) for each website content by the panel of experts rating (n = 8).

Page	Item (Content Heading)	Total Score	I-CVI
Home	myBeST definition (header)	8	1.00
	Who could use myBeST?	8	1.00
	How does myBeST work?	8	1.00
	How was the tool developed?	8	1.00
	Who developed the tool?	8	1.00
	Disclaimer	8	1.00
About	Overview	8	1.00
	Development process	8	1.00
	Our Team	8	1.00
Contact	Contact	8	1.00
		S-CVI/Ave	1.00

myBeST: Malaysian breast cancer survival prognostic tool.

**Table 4:** The face validation index (FVI) for each item (n = 20).

Items	Total Score	FVI
**Prognostic tool:**		
Clarity of the instructions given	20	1.00
Clarity of the input predictors	19	0.95
Understanding the survival prediction output	20	1.00
Appropriateness of the font’s size and type	20	1.00
Appropriateness of the interface layout	20	1.00
**Website content:**		
Understanding the aim of the website	20	1.00
Clarity of the target user for the website	20	1.00
Clarity of the written website content	20	1.00
Appropriateness of the font’s size and type	19	0.95
Appropriateness of the overall interface layout	19	0.95

## Data Availability

The data that support the findings are available from the authors, but restrictions apply to the availability of these data. These data were used under agreement for the current study and are not publicly available. Data are, however, available from the authors but only with the explicit permission of the Director General, Ministry of Health Malaysia.
